# Clinician Perspectives of a Magnetic Resonance Imaging–Based 3D Volumetric Analysis Tool for Neurofibromatosis Type 2–Related Schwannomatosis: Qualitative Pilot Study

**DOI:** 10.2196/71728

**Published:** 2025-07-30

**Authors:** Shelby T Desroches, Alice Huang, Rithvik Ghankot, Steven M Tommasini, Daniel H Wiznia, Frank D Buono

**Affiliations:** 1 Department of Orthopaedics and Rehabilitation, Yale School of Medicine, New Haven, CT, United States; 2 Department of Mechanical Engineering, Yale University, New Haven, CT, United States; 3 Department of Psychiatry, Yale School of Medicine, 300 George Street, New Haven, CT, 06510, United States, 1 2032920980

**Keywords:** neurofibromatosis type 2, vestibular schwannoma, 3D volumetric analysis, magnetic resonance imaging

## Abstract

**Background:**

Accurate monitoring of tumor progression is crucial for optimizing outcomes in neurofibromatosis type 2–related schwannomatosis. Standard 2D linear analysis on magnetic resonance imaging is less accurate than 3D volumetric analysis, but since 3D volumetric analysis is time-consuming, it is not widely used. To shorten the time required for 3D volumetric analysis, our lab has been developing an automated artificial intelligence–driven 3D volumetric tool.

**Objective:**

The objective of the study was to survey and interview clinicians treating neurofibromatosis type 2–related schwannomatosis to understand their views on current 2D analysis and to gather insights for the design of an artificial intelligence–driven 3D volumetric analysis tool.

**Methods:**

Interviews examined for the following themes: (1) shortcomings of the currently used linear analysis, (2) utility of 3D visualizations, (3) features of an interactive 3D modeling software, and (4) lack of a gold standard to assess the accuracy of 3D volumetric analysis. A Likert scale questionnaire was used to survey clinicians’ levels of agreement with 25 statements related to 2D and 3D tumor analyses.

**Results:**

A total of 14 clinicians completed a survey, and 12 clinicians were interviewed. Specialties ranged across neurosurgery, neuroradiology, neurology, oncology, and pediatrics. Overall, clinicians expressed concerns with current linear techniques, with clinicians agreeing that linear measurements can be variable with the possibility of two different clinicians calculating 2 different tumor sizes (mean 4.64, SD 0.49) and that volumetric measurements would be more helpful for determining clearer thresholds of tumor growth (mean 4.50, SD 0.52). For statements discussing the capabilities of a 3D volumetric analysis and visualization software, clinicians expressed strong interest in being able to visualize tumors with respect to critical brain structures (mean 4.36, SD 0.74) and in forecasting tumor growth (mean 4.77, SD 0.44).

**Conclusions:**

Clinicians were overall in favor of the adoption of 3D volumetric analysis techniques for measuring vestibular schwannoma tumors but expressed concerns regarding the novelty and inexperience surrounding these techniques. However, clinicians felt that the ability to visualize tumors with reference to critical structures, to overlay structures, to interact with 3D models, and to visualize areas of slow versus rapid growth in 3D would be valuable contributions to clinical practice. Overall, clinicians provided valuable insights for designing a 3D volumetric analysis tool for vestibular schwannoma tumor growth. These findings may also apply to other central nervous system tumors, offering broader utility in tumor growth assessments.

## Introduction

Neurofibromatosis type 2 (NF2) is an autosomal dominant disorder caused by mutations in the NF2 gene on chromosome 22q12 [1,2]. This disorder is primarily characterized by the growth of vestibular schwannomas (VS), noncancerous brain tumors that arise from Schwann cells along the vestibulocochlear nerve [3]. Depending on the location of VS tumor growth, NF2 patients may develop a range of symptoms, including hearing loss, tinnitus [4], loss of balance, and dizziness [5]. As these tumors progress, they can become life-threatening if they impinge on critical brain structures, such as cranial nerves and the brainstem [6].

Treatment for these tumors typically involves stereotactic radiosurgery, resection surgery [7], or, more recently, Brigatnib, an oral drug showing promising outcomes in those with neurofibromatosis type 2–related schwannomatosis (NF2-SWN) [8]. The decision to pursue these treatments depends on factors such as tumor size, growth, location, and mass, underscoring the importance of accurately monitoring and visualizing tumor progression over time [9]. T1-weighted cranial magnetic resonance imaging (MRI) with intravenous gadolinium has proven effective in visualizing VS tumors in cerebral space [10]. This imaging technique also helps distinguish VS tumors from other anomalies, including cysts, meningiomas, aneurysms, and facial nerve schwannomas, supporting more informed clinical decision-making [11].

Tumor progression can be monitored using various metrics, with the change in tumor size being the most common. In current standard clinical practice, linear analysis involves measuring the largest tumor diameter in two dimensions, usually in an axial or coronal view. Although linear measurements are derived from a patient’s MRI planar slices, the complex geometry of VS tumors can introduce uncertainty in these measurements [7,9]. Larger VS tumors develop characteristic features that may not be fully captured in linear measurements, such as the round body and tail (the “ice cream cone” geometry) [12].

Approximate volumetric analysis addresses some of the limitations of linear analysis by incorporating an extra dimension from 2D to 3D. By approximating the tumor as an ellipsoid in each MR scan slice, the volume of the tumor can be approximated by summing the total areas derived from each slice. While this measure has been shown to be more sensitive to tumor growth than 2D measurements, it has also been found to overestimate tumor growth. As VS tumors continue to develop into the cerebellopontine angle, their shape in each MR slice begins to deviate from that of an ellipsoid [13].

The 3D volumetric analysis addresses the limitations of 2D linear and approximate volumetric methods by calculating tumor volume through the summation of tumor areas from each MR slice. Unlike approximating the tumor as an ellipsoid in each slice, this technique involves directly segmenting the tumor borders, which improves the specificity of the tumor’s boundaries [14]. Although 3D volumetric analysis yields accurate tumor size measurements, it requires manual segmentation, a time-consuming process that can take hours per tumor, necessitating the use of automated volumetric analysis tools [15]. Furthermore, for 3D volumetric analysis to have utility in clinical practice, the 3D visualization resulting from this analysis, which describes the digital 3D model of the segmented tumor in 3D space, must be easily interpretable for clinicians to understand where the tumor exists with reference to the 3D mapping of the patient’s brain.

The 3D modeling technologies such as OsiriX Lite (version 7.0.3; Pixmeo SARL), ITK-Snap (version 2.4.0; ITK-Snap), and BrainLab iPlan (version 4.5; BrainLab) have been described in the literature as performing semiautomated 3D volumetric analysis of VS tumors [16]. While these technologies show promising results with low coefficients of variability, they fail to provide adequate visualization of the segmented tumors in 3D space, limiting their clinical utility. Specifically, these technologies do not have the capabilities to overlay 2D planes on the 3D segmented tumor to demonstrate where different sections of the tumor are located within the patient’s brain scan. Additionally, these technologies do not have features that allow for the overlaying of segmented tumors between chronological scans to investigate areas of tumor growth. We envision that the incorporation of visualization features like these has the potential to broaden the clinical utility of 3D volumetric analysis and visualization software and reduce the need for advanced 3D reconstruction when viewing 3D segmented tumors.

To make 3D volumetric analysis adopted more widely in clinical settings, a thorough understanding of clinical needs related to the analysis methodology (volumetric vs linear) and the 3D visualization must exist. Therefore, the aim of this study was to gain a deeper understanding of clinicians’ current methods for measuring tumor growth, their recommendations for key features in a potential 3D volumetric analysis tool (to be developed), and their concerns regarding how the tool would be clinically integrated. To achieve this, we conducted surveys and interviews with clinicians who treat NF2. We hypothesized that insights from these experienced clinicians would guide the development of a US Food and Drug Administration–regulated auto-segmentation and visualization software to improve the diagnosis and measurement of VS tumors.

## Methods

### Study Sample

This study involved surveys and interviews with clinicians specializing in the treatment and diagnosis of NF2-related schwannomatosis. To be eligible for inclusion, participants had to be clinicians directly involved in the care of NF2-SWN patients and willing to complete a survey or participate in an interview. NF2-SWN patients were not included, as the software is intended primarily as a clinical tool rather than a visualization tool for patients. Clinicians with direct experience in diagnosing and treating NF2-SWN across various specialties, including neurology, neuro-oncology, neurosurgery, neuroradiology, and pediatrics, whose names were found on the Children’s Tumor Foundation website were recruited for this study. Upon identifying clinicians’ names and contact information, emails were subsequently sent to these clinicians, outlining the proposed 3D segmentation software and inviting them to participate in a survey and an interview.

### Ethical Considerations

Ethics approval was obtained from the Yale University Institutional Review Board (approval number: 2000034426) through an exempt review. Verbal informed consent was obtained from all interviewed and surveyed participants before the collection of data, and the institutional review board waived the need for written consent. The clinicians providing the data were deidentified but were not anonymous. No vulnerable populations were included in the data collection of this study. Additional consent was not necessary for any secondary analyses of the collected data. No compensation was provided for participation in this study.

### Survey Data Collection and Analysis

Surveys were conducted via Qualtrics and included 25 statements organized into eight categories as follows: (1) linear/volumetric analysis, (2) markers of VS tumor progression, (3) surgical planning for 2D MRI scans of VS tumors, (4) 3D volumetric analysis, (5) 3D visualization of VS tumors, (6) surgical planning using 3D models of VS tumors, (7) postintervention monitoring, and (8) general questions (Textbox 1). The survey questions were structured as a Likert scale questionnaire, where clinicians indicated their level of agreement with each statement on a scale of 1 to 5, ranging from “completely disagree” to “completely agree.” Additionally, clinicians were asked demographic questions about their clinical specialties, years of experience, and the number of VS cases they had treated.

Textbox 1. **A total of 25 total statements were addressed in both the interviews and in the questionnaire. In the surveys, all clinicians selected 1 out of 5 choices based on how much they agreed with each statement. In the interviews, clinicians had the opportunity to share open-ended responses to the statements.**

**Category 1: 2D linear/volumetric analysis**

1. Linear analysis of vestibular schwannoma (VS) tumors reflects tumor size

2. Volumetric analysis of tumors reflects tumor size

3. Linear measurements are sensitive to small changes in tumor diameter

4. Two different clinicians can review the same magnetic resonance imaging (MRI) but calculate two different tumor sizes.

5. The orientations of bi- and uni-dimensional linear measurements can change from one MRI to the next for a single patient.

6. Patient position in MRI machines influences the orientations of MRI scans, affecting linear measurements.

7. It can be difficult to make a decision about tumor growth from a series of linear measurements due to the lack of clear progression thresholds.

**Category 2: markers of VS tumor progression**

8. Change in tumor size is the most important marker of VS tumor progression

9. Edema and mass effects on surrounding critical structures are good markers of VS tumor progression.

**Category 3: surgical planning with 2D MRI scans of VS tumors**

10. It is simple to determine the best approach to tumor resection before operation (location or angle of entry, how to best maximize extent of resection while avoiding functional areas) from 2D MRI scans.

11. The cranial nerves and blood vessels that need to be avoided during intervention can easily be determined through analysis of MRI scans.

**Category 4: 3D volumetric analysis**

12. 3D volumetric measurements would be more helpful in determining clearer thresholds of tumor growth than linear measurements.

13. 3D volumetric analysis would standardize measurements by reducing variation in measurements of tumor size taken by different clinicians.

14. 3D volumetric analysis would lead to more informed clinical decision-making.

**Category 5: 3D visualization of VS tumors**

15. 3D volumetric measurements must be combined with 3D visualization of the VS tumor to be clinically meaningful.

16. The ability to interact with 3D models of VS tumors in the cerebral space (rotate, zoom, adding or removing structures from view) would provide information helpful for surgical planning.

17. Growth in tumor boundaries observed by overlaying 3D models of VS tumors from MRI scans collected over time is easier to interpret than comparing the same MRI scans in 2D.

18. 3D visualization of VS tumors and their location relative to critical brain structures (cranial nerves, brainstem, etc.) would be helpful in patient care.

19. Knowing regions of rapid versus slow tumor growth on VS tumors would be helpful in treatment planning.

**Category 6: surgical planning with 3D models of VS tumors**

20. 3D visualization of VS tumors in the surrounding cerebral space (relative to critical brain structures) would aid in surgical planning.

21. 3D models of VS tumors would lead to more informed decisions of the type of intervention to perform (eg, different approaches of tumor resection and gamma knife)

**Category 7: postintervention monitoring**

22. Being able to distinguish between post-op changes and residual tumor progression would significantly improve clinical decision-making (ex. After gamma knife).

23. Automatic diagnosis of tumor progress, regression/response, or stability according to changes in size over time would aid treatment planning.

**Category 8: general**

24. Having the ability to forecast tumor growth will significantly improve clinical decision-making.

25. 3D visualizations of VS tumors would significantly improve patient education by allowing patients to see their tumors in 3D space.

In the end, individual survey statement responses were statistically analyzed and means and SD were reported. Higher means indicated that clinicians agreed more with the statement. Following statistical analysis, survey statements relating to tangible features or capabilities of the software were grouped together, and the top four features that clinicians favored based on mean agreement levels were determined.

### Interview Data Collection and Analysis

Two members of the research team (AH and DW) with prior training in conducting semistructured interviews and focus groups jointly facilitated each focus group discussion (FGD) session; the senior author (FB) conducted all the individual interviews. The topics discussed within these discussions mirrored the 25 survey statements reframed as open-ended questions (Textbox 1). The FGD sessions lasted approximately one hour, and the individual interviews lasted approximately 30 minutes. Audio recordings from the FGDs and individual interviews were then transcribed verbatim by Zoom’s transcription feature and returned to the research team for proofreading and subsequent analysis.

The data analysis team (AH, DW, and FB) met regularly to develop the codebook and note potential emergent themes. Interview sessions and data analysis continued simultaneously and iteratively until the team determined that data saturation had been reached [17]. Beginning with an initial set of codes derived from the domains included in the Interview Guide, the coding team members independently coded each transcript to identify potential additional codes. The team then discussed the merits of each existing or new code and created definitions for each for inclusion in the final version of the codebook. The codes were inductively developed, grounded in the data, iteratively reviewed, and revised based on the team’s discussion of additional independently coded transcripts. The total interobserver agreement of the reviewers was assessed over the course of the reviews. The overall total interobserver agreement was 92% across the study.

The codes assigned to each quote were then reviewed for a final time by the first author as part of entering the coded transcripts using Dedoose. Using thematic analysis, the team identified patterns across the entire data set to arrive at a final, parsimonious set of themes. The analysis also examined the data for any “negative” instances where the data did not fit the domains or themes or where no information on specific topics queried was obtained. In addition to the qualitative analysis, descriptive statistics were generated to describe the study sample.

## Results

### Study Sample

A total of 14 clinicians were surveyed from the neurosurgery (n=5), neurology (n=7), oncology (n=1), and general pediatrics (n=1) departments. Clinicians came from 4 different medical institutions and had a range of general clinical experience ranging from 3 to 41 years (mean 21.9, SD 12.1). The average number of VS cases that clinicians treated each year ranged from 0 to 100 (mean 24.6, SD 28.9). The clinicians who did not directly treat VS cases still possessed considerable knowledge about VS treatment through their experience working with NF2-SWN patients.

12 additional clinicians from the neurosurgery (4) neuroradiology (4), neurology (2), and neuro-oncology (2) departments were interviewed to provide open-ended responses to the survey statements. This number was determined based on the amount of interviews needed to reach saturation [17].

### Survey Data Collection and Analysis

The results for all survey statements are reported in Table 1.

**Table 1.** From the Likert questionnaire, clinicians voted with how much they agreed with each statement by choosing (1) “Completely disagree”, (2) “Mostly disagree”, (3) “Neither agree nor disagree”, (4) “Mostly agree”, or (5) “Completely agree”. For the statistical analysis, these metrics of agreement were given numerical values 1 through 5, with 1 aligning with “Completely disagree” and 5 aligning with “Completely agree.”

Statements	Values, mean (SD)
Category 1: 2D linear/volumetric analysis	
Linear analysis of vestibular schwannoma (VS) tumors reflects tumor size.	3.50 (0.94)
Volumetric analysis of tumors reflects tumor size.	4.43 (0.51)
Linear measurements are sensitive to small changes in tumor diameter.	2.43 (1.22)
Two different clinicians can review the same MRI^a^ but calculate two different tumor sizes.	4.64 (0.49)
The orientations of bi- and uni-dimensional linear measurements can change from one MRI to the next for a single patient.	4.21 (0.97)
Patient position in MRI machines influences the orientations of MRI scans, affecting linear measurements.	4.71 (0.47)
It can be difficult to make a decision about tumor growth from a series of linear measurements due to the lack of clear progression thresholds.	3.79 (1.25)
Category 2: markers of VS tumor progression	
Change in tumor size is the most important marker of VS tumor progression	3.50 (0.94)
Edema and mass effects on surrounding critical structures are good markers of VS tumor progression.	4.00 (0.78)
Category 3: surgical planning with 2D MRI scans of VS tumors	
It is simple to determine the best approach of tumor resection before operation (location or angle of entry, how to best maximize extent of resection while avoiding functional areas) from 2D MRI scans.	3.25 (1.16)
The cranial nerves and blood vessels that need to be avoided during intervention can easily be determined through analysis of MRI scans.	3.13 (0.99)
Category 4: 3D volumetric analysis	
3D volumetric measurements would be more helpful in determining clearer thresholds of tumor growth than linear measurements	4.50 (0.52)
3D volumetric analysis would standardize measurements by reducing variation in measurements of tumor size taken by different clinicians.	4.21 (0.58)
3D volumetric analysis would lead to more informed clinical decision making	4.14 (0.66)
Category 5: 3D visualization of VS tumors	
3D volumetric measurements must be combined with 3D visualization of the VS tumor to be clinically meaningful.	3.07 (0.92)
The ability to interact with 3D models of VS tumors in cerebral space (rotate, zoom, adding or removing structures from view) would provide information helpful for surgical planning.	3.86 (1.17)
Growth in tumor boundaries observed by overlaying 3D models of VS tumors from MRI scans collected over time is easier to interpret than comparing the same MRI scans in 2D	4.00 (0.68)
3D visualization of VS tumors and their location relative to critical brain structures (cranial nerves, brainstem, etc.) would be helpful in patient care.	4.36 (0.74)
Knowing regions of rapid versus slow tumor growth on VS tumors would be helpful in treatment planning.	3.71 (0.91)
Category 6: surgical planning with 3D models of VS tumors	
3D visualization of VS tumors in the surrounding cerebral space (relative to critical brain structures) would aid in surgical planning.	3.20 (1.30)
3D models of VS tumors would lead to more informed decisions of the type of intervention to perform (ex. Different approaches to tumor resection, gamma knife)	3.00 (1.41)
Category 7: postintervention monitoring	
Being able to distinguish between post-op changes and residual tumor progression would significantly improve clinical decision-making (eg, after gamma knife).	4.29 (0.73)
Automatic diagnosis of tumor progress, regression/response, or stability according to changes in size over time would aid treatment planning	3.86 (0.66)
Category 8: general	
Having the ability to forecast tumor growth will significantly improve clinical decision-making.	4.77 (0.44)
3D visualizations of VS tumors would significantly improve patient education by allowing patients to see their tumors in 3D space.	3.92 (0.76)

a MRI: magnetic resonance imaging.

#### Category 1: 2D Linear/Volumetric Analysis

Statistical analyses of statement responses revealed that clinicians felt that 3D volumetric analyses (mean 4.43, SD 0.51) offered a slightly greater reflection of tumor size than 2D linear analyses (mean 3.50, SD 0.94). Clinicians’ responses additionally revealed that 2D analyses were generally not sensitive to small changes in tumor growth (mean 2.43, SD 1.22). Statements addressing variability in linear measurements highlighted a consensus around the potential for inconsistency due to factors like clinician interpretation (mean 4.64, SD 0.49), MRI orientation (mean 4.21, SD 0.97), and patient orientation (mean 4.71, SD 0.47), with high agreement that these factors impact linear measurements. Additionally, there was moderate agreement (mean 3.79, SD 1.25) surrounding the statement that linear measurements were difficult to interpret.

#### Category 2: Markers of VS Tumor Progression

Clinicians generally agreed that secondary effects, such as edema and impact on surrounding structures, serve as valuable markers for tracking VS tumor progression (mean 4.0, SD 0.78) and demonstrated a moderate agreement that tumor size is the most important marker above these secondary effects (mean 3.5, SD 0.94). This demonstrated the opinion that all metrics, including tumor size and secondary effects, may hold relatively equal importance to clinicians.

#### Category 3: Surgical Planning With 2D MRI Scans of VS Tumors

Responses indicated skepticism about the utility of 2D MRI scans in preoperative planning, with clinicians leaning towards neither disagreeing nor agreeing that these scans alone allow clear determination of surgical approach (mean 3.25, SD 1.16) and that critical structures can easily be determined through 2D MRI analyses (mean 3.13, SD 0.99).

#### Category 4: 3D Volumetric Analysis

Clinicians supported the use of 3D volumetric analysis over linear measurements, suggesting it would enhance consistency across clinicians (mean 4.21, SD 0.58), help establish clearer growth thresholds (mean 4.50, SD 0.52), and improve clinical decision-making (mean 4.14, SD 0.66).

#### Category 5: 3D Visualization of VS Tumors

In terms of features important for a 3D visualization software, most clinicians somewhat agreed that interactions with 3D models (rotating, zooming, or hiding structures) (mean 3.86, SD 1.17) and knowing areas of rapid versus slow growth (mean 3.71, SD 0.91) would be important for treatment planning. Clinicians were overall in agreement that overlaying chronological tumors to visualize growth (mean 4.00, SD 0.68) and viewing tumors relative to critical brain structures (mean 4.36, SD 0.74) were important for understanding tumor growth and improving patient care. However, clinicians generally neither agreed nor disagreed that visualizations were necessary for 3D analyses to be clinically useful (as opposed to volumes alone) (mean 3.07, SD 0.92).

#### Category 6: 3D Visualization of VS Tumors

In general, clinicians did not strongly agree or disagree regarding statements that claimed that 3D visualization would be a helpful aid for surgical planning (mean 3.20, SD 1.30) and would help make more informed clinical decisions (mean 3.00, SD 1.41).

#### Category 7: Postintervention Monitoring

Clinicians somewhat agreed that automatic diagnosis of tumor changes (regression/response or stability) according to changes in size would aid in treatment planning (mean 3.86. SD 0.66).

There was a greater agreement that the ability to distinguish between postoperative changes and residual tumor progression through chronological tumor overlaying would improve clinical decision-making (mean 4.29, SD 0.73)

#### Category 8: General

Clinicians strongly agreed that the ability to forecast tumor growth would be a significant improvement in clinical decision-making (mean 4.77, SD 0.44). In addition, clinicians somewhat agreed that 3D visualizations would be helpful for patient education (mean 3.92, SD 0.76).

The top four features that clinicians felt would be most beneficial in a 3D analysis and visualization software are listed in Textbox 2. These four features are listed in the order of priority based on the mean agreement levels in Table 1.

Textbox 2. **List of 4 main features, ranked in order of clinician’s priority, for 3D tumor visualization interface.**

1. Forecast tumor growth based on previous 3D growth trends
2. Visualization of tumors with reference to other critical brain structures
3. Overlaying chronological 3D tumor models from the same patient to visualize growth
4. Interacting with 3D models, including rotating, zooming, and adding or removing tumors from view.

### Interview Data Collection and Analysis

Interview findings were analyzed and sorted into four corresponding themes as follows: (1) shortcomings of the currently used linear analysis, (2) utility of 3D visualizations, (3) features of an interactive 3D modeling software, and (4) lack of a gold standard to assess the accuracy of 3D volumetric analysis.

#### Theme 1: Shortcomings of the Currently Used Linear Analysis

Clinicians observed that most medical professionals tracking the size or growth of VS tumors rely on linear analysis, primarily using uni- or bi-dimensional measurements. As such, linear analysis is considered the current “gold standard.”

In general, discussions on the limitations of current linear analysis methods generally focused on the variability in measurements when conducted at different times or by different individuals. They highlighted several factors contributing to variability in these measurements. First, there is a lack of standardization in the linear analysis procedure, leading to variability in how clinicians perform measurements. Additionally, many clinicians will perform linear analysis by first determining the longest tumor diameter slice, a process that is subjective. Clinicians also noted that variations in patient orientation during MRI scans can result in inconsistent tumor presentations in axial and coronal views across different scans. Consequently, comparing linear measurements from one scan to the corresponding slice in another may be inaccurate. Finally, they emphasized that due to the subjectivity and potential inaccuracies in these measurements, tumor growth is more easily detected in scans taken over longer intervals, while frequent scans make it harder to discern changes.

#### Theme 2: Utility of 3D Visualizations

The clinicians interviewed agreed that visualizing VS tumors in relation to critical brain structures would significantly improve diagnosis and treatment planning. Currently, 2D MRI images must be mentally reconstructed by clinicians to understand how tumors interact with these critical structures in a 3D space. This mental reconstruction requires an abstract and advanced understanding of 3D cerebral anatomy, which typically comes with experience.

Clinicians further suggested that creating 3D models of tumors, along with color gradients to represent tumor growth over time, could enhance the visualization of tumor progression and help bridge the knowledge gap between experienced and less experienced clinicians. They also noted that additional markers—such as edema, necrotic core growth, and mass effects like compression on surrounding brain structures—are important in assessing tumor progression. Several clinicians emphasized that segmenting and visualizing these markers in 3D, alongside the tumor itself, could provide critical information for optimizing tumor management.

#### Theme 3: Features of an Interactive 3D Modeling Software

The clinicians agreed that adding interactive features to 3D modeling software would enable a more comprehensive understanding of each patient’s case compared with simply viewing static 3D models on a screen. They suggested several interactive capabilities, including (1) adjusting the transparency of tumor models, (2) zooming in and out, and (3) rotating the models. These interactive functions were seen as providing significantly more information than traditional static 2D imaging. Clinicians also proposed incorporating animations of tumor growth over time, with newer tumors overlaid on older ones, to improve clinical understanding of disease progression. Additionally, they recommended incorporating a coordinate grid within the 3D tumor model to aid in tumor localization within the cerebral space, which could assist in guiding stereotactic interventions.

#### Theme 4: Lack of a Gold Standard to Assess the Accuracy of 3D Volumetric Analysis

In addition to providing suggestions regarding features that should be included in 3D visualization software, clinicians shared concerns regarding the incorporation of this type of software into clinical practice. These concerns primarily surrounded the novelty of this approach and the difficulty of validating measurements.

The clinicians interviewed noted that because linear measurements are currently the most common metric of tumor size, most experienced clinicians are adept at interpreting linear measurements to determine tumor management and treatment. By combining these linear measurements with the Response Evaluation Criteria in Solid Tumors (RECIST), clinicians can evaluate tumor growth. Thus, in the interviews, the clinicians expressed the concern that due to the novelty of using volume as a metric of tumor size, they may be unfamiliar with the clinical implication of these measurements and thus may be unable to take advantage of the additional information this 3D tool provides. Furthermore, clinicians expressed concerns regarding the lack of a gold standard by which to assess the accuracy of volumetric measurements. While no gold standard exists to determine the accuracy of linear measurements, the novelty of volumetric analysis necessitates an additional metric of accuracy.

## Discussion

### Principal Findings

In this study, surveys and clinical interviews were conducted to evaluate the efficacy of current 2D tumor analysis protocols and to assess the clinical interest, recommendations, and concerns regarding the incorporation of 3D volumetric analysis for the treatment of NF2-related schwannomatosis. In summary, the consensus among clinicians was that a 3D volumetric analysis and visualization tool could greatly benefit the treatment of NF2-related schwannomatosis, but it must be implemented with caution. We believe that these clinician perspectives are extremely important as 3D volumetric analyses are increasingly being discussed within the literature, and for these technologies to have clinical relevance, the perspectives of clinicians must be at the forefront.

Clinicians were enthusiastic about the prospect of using 3D volumetric analysis in lieu of traditionally used linear analysis. This was largely due to several shortcomings of linear analysis that were highlighted through both survey results and interviews, with the two main concerns being varying patient orientations during MR imaging procedures causing variability in 2D image presentation and the subjectivity of defining the maximum tumor diameter in linear assessments. Despite efforts to collect linear measurements across scans as uniformly as possible by locating anatomical landmarks for individual patients, it can be challenging to ensure the measurement is collected in the same location. These limitations described by the interviewed clinicians align with those described by the Response Evaluation in Neurofibromatosis and Schwannomatosis consortium, something that led to their recommendation of using 3D volumetric analysis to assess tumor size initially [18].

From the interview and survey findings, there were several features of 3D analysis and visualization software that were believed to have the potential to benefit clinical practice. From interviews, the main consensus was that the ability to visualize the relationship between critical brain structures and tumors in 3D space would be the largest benefit of a 3D analysis and visualization tool as it would reduce the need for 3D mental reconstruction of where the tumor exists within 3D space. This feature was largely supported by the survey results as well, with “visualizing tumors with respect to critical brain structures” being the second highest feature that clinicians believed would be beneficial (Textbox 2).

Other features highlighted in both surveys and interviews demonstrated that the software should be interactable by incorporating zoom and rotation, allow for the adding/removing of the tumor from view, and have transparency adjustment features. These features are even more important when paired with the capability to view multiple chronological tumors at once, one of the other main features identified by both interviewed and surveyed clinicians as being important. Specifically, clinicians expressed enthusiasm for the prospect that these chronological tumor overlays could eventually lead to the capability of forecasting tumor growth. In Textbox 2 this “forecasting feature” ranked as the highest priority feature for clinicians demonstrating a belief that this could have a major positive impact on clinical treatment. Overall, the clinical opinions regarding important features demonstrate that current 3D modeling technologies such as OsiriX Lite, ITK-Snap, and BrainLab iPlan may not be heavily integrated into clinical practice due to shortcomings in their visualization capabilities, specifically their inability to overlay chronological tumors. Jester et al [19] provide a visualization of this chronological tumor overlay, demonstrating how it can give clinicians an interpretable vision of tumor growth over time.

Some statements regarding the benefits or potential use of 3D volumetric analysis or 3D visualizations were met with hesitancy, with average agreement levels nearing 3.0 (neither disagree nor agree). These statements claimed that 3D volumetric measurements must be combined with visualization to be clinically meaningful (mean 3.07, SD 0.92), that 3D models of VS tumors would lead to more informed decisions about the type of intervention needed (mean 3.00, SD 1.41) and that 3D visualization of VS tumors in the surrounding cerebral space would aid in surgical planning (mean 3.20, SD 1.30). This hesitancy demonstrates that while visualizations are important to clinicians, more precise volumes to compare between chronological scans may still be more important. Additionally, the hesitancy surrounding the use of 3D visualization to better define treatment approaches could also be attributed to a lack of understanding of how these visualizations may be used in this setting, something that could be better demonstrated to clinicians with a more detailed overview of the software’s design.

Despite recommendations to move towards volumetric analyses, the interviewed clinicians also expressed concerns about how they would determine the clinical implications of volume measurements. These clinicians felt that they could comfortably determine the clinical implications of linear measurements by assessing the trends of uni- and bi-dimensional linear measurements of tumor size, but they lacked a similar knowledge of trends for volumetric measurements. As a future step to increase clinical confidence in the interpretation of tumor volumes, correlation studies can be conducted to determine the approximate relationship between linear and volumetric measurements. Additionally, future development of this diagnostic software can integrate linear analysis measurements as well, automating assessments of uni- and bi-dimensional diameters. Through such studies, clinicians can gain exposure to using segmentation software to perform volumetric analysis and interpret volume measurements while collecting familiar linear measurements. Pairing these correlation studies with currently used RECIST criteria can encourage the adoption of 3D volumetric analysis in the clinical space.

For full adoption and validation of 3D volumetric analysis in the clinical space, multi-center validation studies must take place with clinicians from multiple institutions performing 3D volumetric analysis on a diverse array of MRIs. DICE scores can be used as a metric to evaluate the differences in voxel selection within each scan between clinicians. To generate RECIST-compatible standards, current RECIST protocols should be used as a basis to form corresponding 3D-specific protocols. This will require collaboration from multi-institutional research and clinical communities interested in the full adoption of 3D volumetric analysis.

### Limitations

Clinical interviews incorporated in this study were conducted with a limited group of clinicians from just four institutions. Incorporating clinical feedback from a larger and broader group of clinicians experienced in the diagnosis and treatment of NF may more clearly elucidate the strengths and limitations of a novel segmentation and visualization software. Specifically, including perspectives from clinicians from institutions with varying levels of expertise, resources, and knowledge in treating NF and understanding how these varied departmental structures impact perspectives was not done in this study, but could aid in generating a tool more applicable to all clinicians, not just those from high-resource medical institutions. Additionally, though the sole user of this software is intended to be clinicians, other users, such as researchers, may use this software. However, within this study, interviews were limited to just clinicians, potentially limiting the information gathered.

### Conclusions

Clinicians were overall in favor of the adoption of 3D volumetric analysis techniques for measuring VS tumors but expressed concerns regarding the novelty and inexperience surrounding these techniques. However, clinicians felt that the ability to visualize tumors with reference to critical structures, to overlay structures, to interact with 3D models, and to visualize areas of slow versus rapid growth in 3D would be valuable contributions to clinical practice. Overall, clinicians provided valuable insights for designing a 3D volumetric analysis tool for VS tumor growth. These findings may also apply to other central nervous system tumors, offering broader utility in tumor growth assessments.

## Abbreviations

MRI: magnetic resonance imaging
NF2: neurofibromatosis type 2
NF2-SWN: neurofibromatosis type 2–related schwannomatosis
RECIST: Response Evaluation Criteria in Solid Tumors
VS: vestibular schwannoma

## References

[1] Asthagiri AR, Parry DM, Butman JA (2009). Neurofibromatosis type 2. Lancet.

[2] Starnoni D, Giammattei L, Cossu G (2020). Surgical management for large vestibular schwannomas: a systematic review, meta-analysis, and consensus statement on behalf of the EANS skull base section. Acta Neurochir (Wien).

[3] Slattery WH (2015). Neurofibromatosis type 2. Otolaryngol Clin North Am.

[4] Halliday D, Emmanouil B, Pretorius P (2017). Genetic severity score predicts clinical phenotype in NF2. J Med Genet.

[5] Carlson ML, Link MJ (2021). Vestibular schwannomas. N Engl J Med.

[6] Anderson JL, Gutmann DH (2015). Neurofibromatosis type 1. Handb Clin Neurol.

[7] Nguyen D, de Kanztow L (2019). Vestibular schwannomas: a review. Appl Radiol.

[8] Plotkin SR, Yohay KH, Nghiemphu PL (2024). Brigatinib in NF2-*r*elated schwannomatosis with progressive tumors. N Engl J Med.

[9] Selleck AM, Rodriguez JD, Brown KD (2021). Vestibular schwannoma measurements-is volumetric analysis clinically necessary?. Otol Neurotol.

[10] Jackler RK, Shapiro MS, Dillon WP, Pitts L, Lanser MJ (1990). Gadolinium-DTPA enhanced magnetic resonance imaging in acoustic neuroma diagnosis and management. Otolaryngol Head Neck Surg.

[11] Lin EP, Crane BT (2017). The management and imaging of vestibular schwannomas. AJNR Am J Neuroradiol.

[12] Lees KA, Tombers NM, Link MJ (2018). Natural history of sporadic vestibular schwannoma: a volumetric study of tumor growth. Otolaryngol Head Neck Surg.

[13] Ho HH, Li YH, Lee JC (2018). Vestibular schwannomas: accuracy of tumor volume estimated by ice cream cone formula using thin-sliced MR images. PLoS One.

[14] Morris KA, Parry A, Pretorius PM (2016). Comparing the sensitivity of linear and volumetric MRI measurements to detect changes in the size of vestibular schwannomas in patients with neurofibromatosis type 2 on bevacizumab treatment. Br J Radiol.

[15] MacKeith S, Das T, Graves M (2018). A comparison of semi-automated volumetric vs linear measurement of small vestibular schwannomas. Eur Arch Otorhinolaryngol.

[16] Kollmann P, Mautner VF, Koeppen J (2020). MRI based volumetric measurements of vestibular schwannomas in patients with neurofibromatosis type 2: comparison of three different software tools. Sci Rep.

[17] Guest G, Bunce A, Johnson L (2006). How many interviews are enough?: An experiment with data saturation and variability. Field Methods.

[18] Dombi E, Ardern-Holmes SL, Babovic-Vuksanovic D (2013). Recommendations for imaging tumor response in neurofibromatosis clinical trials. Neurology (ECronicon).

[19] Jester N, Singh M, Lorr S, Tommasini SM, Wiznia DH, Buono FD (2025). The development of an artificial intelligence auto-segmentation tool for 3D volumetric analysis of vestibular schwannomas. Sci Rep.

